# Hepatocellular Carcinoma After Successful Treatment of Hepatitis C Virus with Ledipasvir/Sofosbuvir Presenting as Acute Pulmonary Tumor Embolism

**DOI:** 10.7759/cureus.4336

**Published:** 2019-03-27

**Authors:** Saad Habib, Mohammed Azam, Abdul Hasan Siddiqui, Kartikeya Rajdev, Michel Chalhoub

**Affiliations:** 1 Internal Medicine, Staten Island University Hospital, Northwell Health, Staten Island, USA; 2 Pulmonary and Critical Care Medicine, Staten Island University Hospital, Northwell Health, Staten Island, USA

**Keywords:** hepatocellular carcinoma, hcv, ledipasvir, pulmonary embolism, sofobuvir

## Abstract

Hepatitis C virus (HCV)-induced cirrhosis is a major cause of hepatocellular carcinoma (HCC) worldwide. HCC is an aggressive malignancy in which tumor thrombus can invade portal vein, hepatic veins and inferior vena cava (IVC) in the later stages. Our case brings to attention, HCV patient population who might need long-term follow-up to ensure HCV clearance. Physicians should ensure appropriate follow-up after treatment of HCV and should emphasize on the ongoing screening for HCC in patients with cirrhosis or advanced fibrosis, regardless of antiviral treatment outcome.

## Introduction

Hepatocellular carcinoma (HCC) is the fourth most common cancer and the third leading cause of cancer-related deaths in the world [1]. HCC is a highly aggressive malignancy in which tumor thrombus can invade portal vein, hepatic veins and inferior vena cava (IVC) in the later stages [2]. Pulmonary tumor emboli are end-stage manifestation of malignancy and have a very poor prognosis [3]. Herein, we present a rare case of HCC successfully treated for hepatitis C virus (HCV) with ledipasvir-sofosbuvir presenting four years later as pulmonary tumor embolism with tumor thrombi in the IVC, right atrium, and right ventricle (RV).

## Case presentation

**Figure 1:**
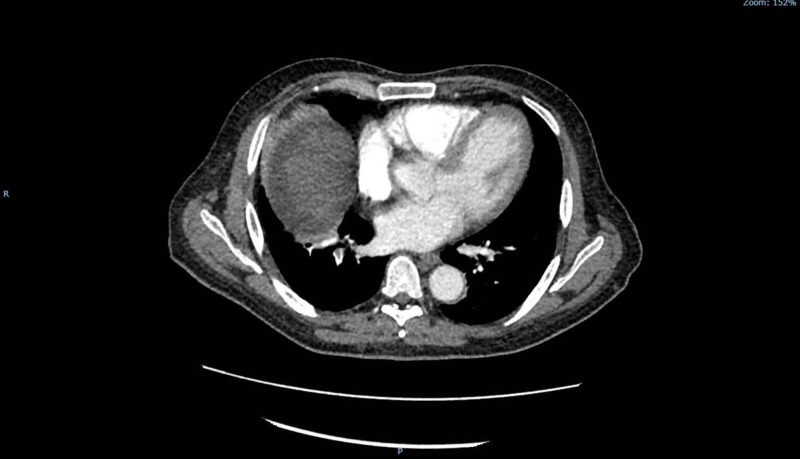
Axial computed tomography of the chest with intravenous contrast showing pulmonary embolism in the right lower lobe of the lung.

**Figure 2:**
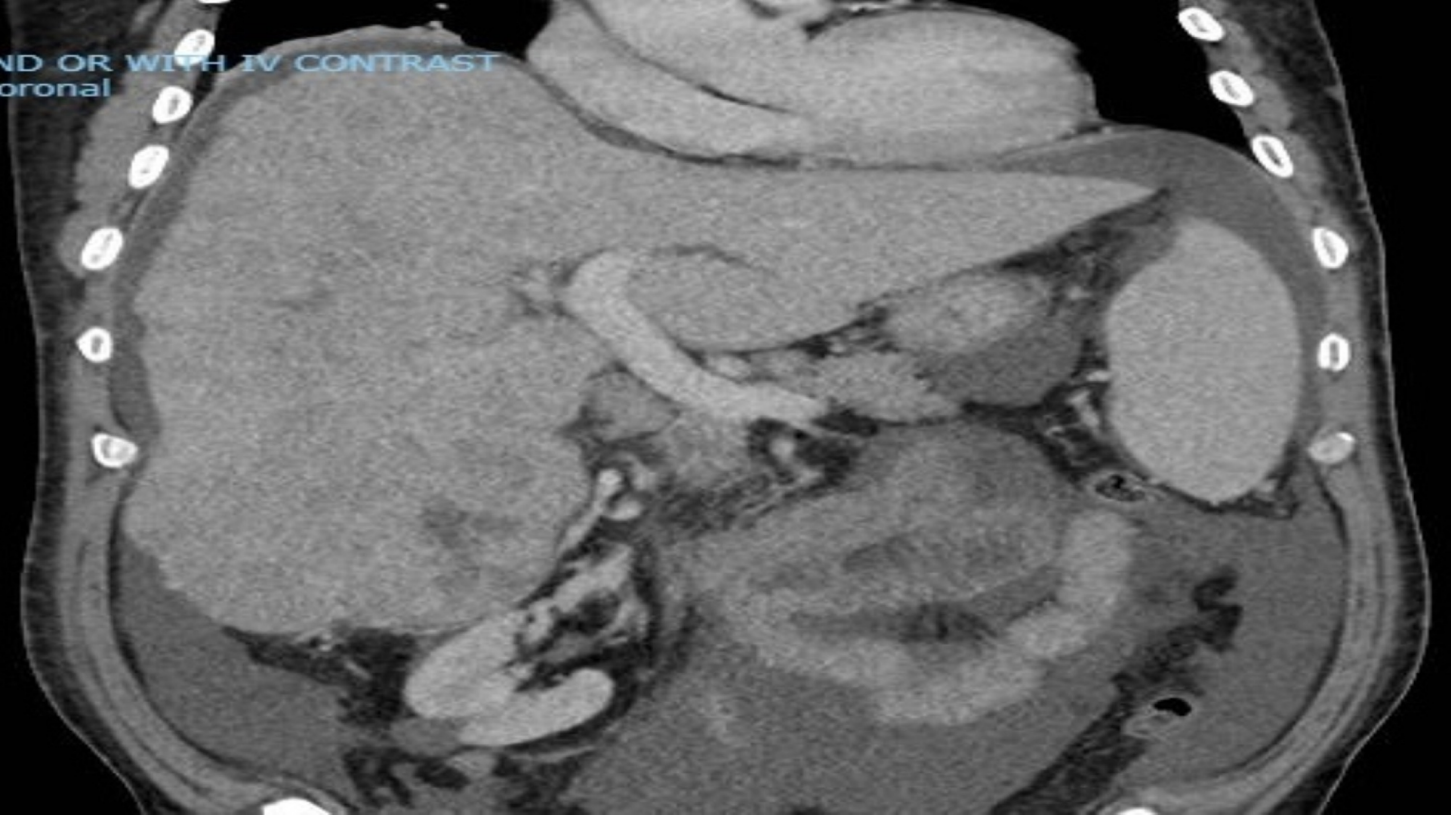
Computed tomography of the abdomen showing a cirrhotic liver with infiltrative tumor.

A 58-year-old male presented to the hospital with chief complaints of dyspnea on exertion associated with pleuritic chest pain and fatigue for two weeks prior to presentation. Medical history was significant for hypertension and hepatitis C genotype 1 treated with ledipasvir/sofosbuvir four years ago. HCV was successfully treated with achievement of sustained virologic response four years ago. The patient did not follow-up after treatment completion. On physical examination, mildly distended, non-tender abdomen was noted. Computed tomography (CT) angiogram of chest was done due to an elevated D-Dimer of 1878 ng/ml and the patient was found to have a segmental pulmonary embolism in the right lower lobe of the lung (Figure 1). The patient was started on anticoagulation for the pulmonary embolism. A duplex ultrasonography of lower extremities was negative for deep vein thrombosis (DVT). In addition, the patient had an abnormal liver profile with an elevated aspartate transaminase (AST) of 416 U/L, alanine transaminase (ALT) of 95 U/L and alkaline phosphate of 474 U/L. The patient had serum albumin level at 3.2 g/dl and total serum bilirubin level at 0.9 mg/dl. Further imaging of the abdomen revealed a cirrhotic liver with infiltrative tumor nearly completely replacing the right hepatic lobe measuring 14.9 x 11.7 x 13.7 cm which was suspicious for HCC (Figure 2). Tumor thrombus within the right portal vein, right hepatic vein extending to the IVC was also revealed. Echocardiogram revealed a possible thrombus (or mass) by the base of the RV appearing as a thrombus in transit. Further workup revealed an elevated alpha-fetoprotein (AFP) of 146 ng/ml. Cancer Antigen 19-9 was elevated to 125 U/ml whereas carcinoembryonic antigen was 1.7 ng/ml which was within normal limits. HCV RNA was detected to 82668 IU/ml. In the patient’s care, multiple sub-specialties were involved and a decision was made to continue anticoagulation due to the overall prothrombotic state of the patient. The patient was deemed not a surgical candidate due to the extent of the cancer and was referred to oncology outpatient for possible plan to start radiotherapy or chemotherapy with Sorafenib. The patient did not follow up with oncology and opted for palliative care, and died in hospice after four months.

## Discussion

HCV-induced cirrhosis is a major cause of HCC worldwide. The redetection of HCV RNA in our patient can either be explained by ledipasvir-sofosbuvir treatment failure with HCV reactivation, HCV relapse or HCV reinfection. The preferred antiviral regimen for the vast majority of patients with chronic HCV infection was ledipasvir-sofosbuvir when our patient was treated [4]. HCV NS5A protein inhibitor, ledipasvir and the NS5B nucleotide polymerase inhibitor, sofosbuvir [5] is highly effective for both treatment-naive and experienced patients with genotype 1 infection, even in the setting of cirrhosis [6]. The goal of treatment is to eradicate HCV RNA, which is predicted by the achievement of a sustained virologic response, defined by the absence of HCV RNA by polymerase chain reaction 12 weeks after finishing treatment [7]. Follow-up after treatment includes checking the viral load 12 weeks after the cessation of therapy to evaluate for a sustained virologic response [8]. Patients with advanced fibrosis or cirrhosis also warrant ongoing screening for hepatocellular carcinoma with liver ultrasonography every six months [9], regardless of antiviral treatment outcome [10]. However, our patient’s HCV was treated successfully but the patient was lost to follow-up after HCV treatment. Virologic failure with ledipasvir-sofosbuvir has been associated with several NS5A mutations that reduce susceptibility to ledipasvir, most commonly Q30R, Y93H/N, and L31M in subtype 1a virus and Y93H in subtype 1b virus [4]. Approximately 20% of genotype 1 viruses harbor polymorphisms that confer reduced susceptibility to ledipasvir [11]. Mutational analysis testing for NS5A mutations is now commercially available. For patients who fail treatment, resistance to ledipasvir was observed more commonly, whereas resistance to sofosbuvir was less common [4].

The diagnosis of HCC is typically made by radiological liver imaging in combination with serum AFP without the need for biopsy especially in patients with cirrhosis [12]. Therefore, in our patient, the diagnosis of HCC was made based on the history of hepatitis C cirrhosis and radiological findings, which were typical of HCC. The clinical manifestations of pulmonary tumor embolism are nonspecific [12] and may present with features suggestive of venous thromboembolism [13] or pulmonary hypertension like dyspnea, hypoxemia, right heart strain, and clear lungs [14]. In HCC patients who have no underlying cardiopulmonary diseases, chest pain and shortness of breath should increase the suspicion for pulmonary tumor embolism [12]. Pulmonary tumor embolism refers to the identification of tumor within pulmonary blood vessels on pathologic lung biopsy samples [3] which can be done with endobronchial ultrasound-guided transbronchial needle aspiration [15] or surgical lung biopsy. There can be two types of IVC thrombus, bland thrombus and tumor thrombus. Bland thrombus is an isolated thrombus, which commonly arise from DVT of lower extremities. Tumor thrombus contains both tumor and thrombotic components and usually extends in the direction of blood flow towards right atrium. Imaging modalities such as endobronchial ultrasound, computed tomography, magnetic resonance imaging and abdominal ultrasonography are useful in rapid detection and follow-up of tumor thrombus [16]. Vascular invasion and tumor throm­bosis formation are prominent characteristics in the majority of cases of advanced HCC. The incidence of hepatic vein thrombosis in HCC patients is 1.4‑4.9%. Tumor thrombosis from HCC invading the hepatic vein occasionally spreads to the IVC and even the right atrium which has been reported in 0.67-3% of HCC patients [12].

Extension of IVC thrombus into right atrium (RA) can present as RA mass [16]. Primary thrombi in the right atrium are usually immobile and attached to the atrial wall whereas secondary thrombi are mobile [12]. In the case of a mobile mass formation in the cardiac cavities, ball valve thrombus syndrome presenting with cardiac murmurs, respiratory distress, syncope, and/or shock causing sudden cardiac death may occur [17]. Echocardiography has been shown to be useful in detection of cardiac extension. Echocardiography provides information about the mobility of tumor thrombus and the relation of valve and cardiac muscle with respect to the thrombus [18]. Transesophageal echocardiography (TEE) is preferred over transthoracic echocardiography (TTE) as subcostal view on TTE may be effected by the hepatic tumor and TEE provides more accurate information not only about the location of mass in relation to atrial wall or tricuspid valve but also the position with respect to superior and inferior vena cava. Furthermore, TEE may detect involvement of inferior vena cava or right atrium which may be missed by TTE. However, TEE maybe difficult to perform in patients with HCC and cirrhosis with esophageal varices [18].

Definitive therapy of pulmonary tumor emboli is directed at treating the primary tumor [3]. Surgical resection or liver transplant for those meeting the Milan criteria is the best therapeutic approach for HCC [18]. However, in patients with HCC complicated with tumor thrombi the standard treatment has not been established, due to its rarity and therapeutic difficulties [19]. In advanced HCC, which is unresectable, a combination of different non-surgical therapeutic approaches such as sorafenib, hepatic arterial infusion chemotherapy, radiotherapy, transarterial chemoembolization (TACE) and radiofrequency ablation may improve survival [18]. Radio frequency ablation and intra-arterial chemoembolization have been shown to be effective for relieving symptoms and prolonging life in few patients. Recently, TACE has become the most popular palliative treatment for patients with unresectable HCC, and portal vein tumor thrombus is no longer considered an absolute contraindication for TACE. In a recent meta-analysis, external beam radiotherapy was found to be a feasible palliative treatment option for HCC patients with IVC and or/RA involvement [19]. Chemotherapeutic agents like sorafenib have also been used in patients with unresectable tumor but preserved hepatic synthetic function (Conference poster presentation: Habib S, Siddiqui AH, Rajdev K, Azam M, Jilani T, Chalhoub M. Acute Pulmonary Embolism Blood Clot or Tumor Embolus?. Society Of Critical Care Medicine; January 2019)*.*

The prognosis of HCC patients with tumor thrombi is extremely poor, with median survival of about three months from diagnosis without treatment [19]. The one‑year survival rate among HCC patients with pulmonary embolism and IVC invasion who received surgery was 40% [12]; however, in the group without surgery, the median survival time was only three days and none of the patients survived for > two months [12]. In the treatment of HCC extending into the IVC/RA, hepatectomy and thrombectomy group had a median survival of 19 months, TACE group had a median survival of 4.5 months [20], and symptomatic treatment had a median survival of five months. This data indicated that surgery (either removing thrombus combined with hepatectomy or only tumor thrombus extraction) might result in better survival when compared with other non-surgical therapies [20]. Sorafenib, a multikinase inhibitor of PDGF, VEGF and threonine kinase (Raf), has been shown to increase survival of patients with advanced HCC by three months [18].

## Conclusions

In conclusion, our case is unique as advanced HCC presented as pulmonary tumor embolism after successful treatment of HCV. In patients with HCC, who present with respiratory distress and chest pain, clinicians should have a high index of suspicion for pulmonary tumor embolism. Our case emphasizes the importance of HCC screening in patients treated for hepatitis C and have advanced fibrosis or cirrhosis for early detection and management of hepatocellular carcinoma.
